# Complementary proteomics strategies capture an ataxin-1 interactome in Neuro-2a cells

**DOI:** 10.1038/sdata.2018.262

**Published:** 2018-11-20

**Authors:** Sunyuan Zhang, Nicholas A. Williamson, Marie A. Bogoyevitch

**Affiliations:** 1 Department of Biochemistry and Molecular Biology, University of Melbourne, Parkville, Victoria 3010, Australia; 2 Bio21 Molecular Science and Biotechnology Institute, University of Melbourne, Parkville, Victoria 3010, Australia

**Keywords:** Protein-protein interaction networks, Proteomics, Neurodegenerative diseases

## Abstract

Ataxin-1 mutation, arising from a polyglutamine (polyQ) tract expansion, is the underlying genetic cause of the late-onset neurodegenerative disease Spinocerebellar ataxia type 1 (SCA1). To identify protein partners of polyQ-ataxin-1 in neuronal cells under control or stress conditions, here we report our complementary proteomics strategies of proximity-dependent biotin identification (BioID) and affinity purification (via GFP-Trap pulldown) in Neuro-2a cells expressing epitope-tagged forms of ataxin-1[85Q]. These approaches allowed our enrichment of proximal proteins and interacting partners, respectively, with the subsequent protein identification performed by liquid chromatography-MS/MS. Background proteins, not dependent on the presence of the polyQ-ataxin-1 protein, were additionally defined by their endogenous biotinylation (for the BioID protocol) or by their non-specific interaction with GFP only (in the GFP-Trap protocol). All datasets were generated from biological replicates. Following the removal of the identified background proteins from the acquired protein lists, our experimental design has captured a comprehensive polyQ-ataxin-1 proximal and direct protein partners under normal and stress conditions. Data are available via ProteomeXchange, with identifier PXD010352.

## Background & Summary

Although the biochemical and biophysical characterization of an isolated protein can assess its activities and regulation, the study of protein-protein interaction (PPI) networks in cells holds the promise to reveal new partnerships and actions that can impact applications as far reaching as drug discovery for different disease scenarios^1,2^. For the late-onset neurodegenerative disease spinocerebellar ataxia type 1 (SCA1), an expansion of the polyglutamine (polyQ) tract of ataxin-1 leads to accumulation of mutant polyQ-ataxin-1 protein in nuclear bodies^3–6^. These nuclear bodies are dynamic protein assemblies^7^; thus, the PPI networks of polyQ-ataxin-1 remain a source of interest^8^, particularly as the intrinsically disordered region of the expanded polyQ region may make significant contributions.

Interacting protein partners can contribute to ataxin-1 stability and/or functions. The best-studied examples include the 14-3-3 family of scaffold proteins, the transcriptional regulator Capicua (CIC), the splicing factor U2AF 65 kDa subunit (U2AF65 or U2AF2) and RNA binding motif protein 17 (RBM17). For the 14-3-3 proteins, their interaction with phosphorylated ataxin-1 in the cell cytoplasm prevents ataxin-1 dephosphorylation and degradation, and inhibits the nuclear translocation of ataxin-1 that is required for its toxicity^9,10^. In contrast, other nuclear protein partners such as CIC, U2AF65 or RBM17 function in transcriptional repression or RNA splicing^3,11,12^. Decreased interactions with CIC or U2AF65, but increased interaction with RBM17, all contribute to polyQ-ataxin-1 toxicity^3,11,12^. Clearly a deeper understanding of ataxin-1 actions and toxicity will require comprehensive assessment of the PPI network that creates the polyQ-ataxin-1 interactome.

Approaches previously implemented to identify the polyQ-ataxin-1 interactome have included yeast-two-hybrid screening or affinity purification of ataxin-1 complexes. Yeast-two-hybrid screening with ataxin-1[82Q] identified multiple potential ataxin-1 binding partners^13^. However, the use of yeast as host in these screens can compromise post-translational modifications that modulate ataxin-1’s PPI network^14^. An alternative approach executed within a mammalian cell context, has been the purification of GFP-ataxin-1[82Q] from transfected non-neuronal (HEK293) cells followed by mass spectrometry to identify co-associating proteins^15^. However, this capture of protein complexes can be biased towards stable PPI rather than capturing a dynamic ataxin-1 interactome. Therefore, additional proteomics approaches within a neuronal cell system are expected to yield a broader but more relevant repertoire of protein partners for polyQ-ataxin-1 to aid understanding of SCA1 disease pathogenesis.

**Figure 1:**
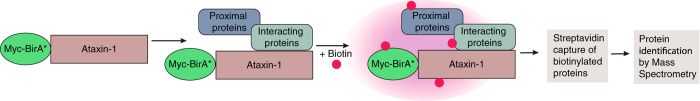
Principle of BioID screening of ataxin-1 proximal or interacting partners. The recombinant protein comprised of ataxin-1[85Q] fused to BirA* biotin ligase enables the biotinylation of directly-interacting or proximal proteins followed by capture on streptavidin-agarose, purification and mass spectrometry analysis for protein identification.

When dynamic and transient PPI maintain protein assemblies, partner identification is a significant challenge. However, the implementation of proximity-dependent biotin identification (BioID) has made significant advances^16^, particularly for a number of dynamic protein complexes^17–20^. Knowing that mutant ataxin-1 protein forms dynamic nuclear bodies, we adopted the BioID approach, fusing the BirA* mutant biotin ligase to ataxin-1[85Q], and thus developing a system in which ataxin-1 proximal or interacting proteins will be biotinylated and subsequently captured by interaction with streptavidin-agarose for mass spectrometry analyses (Fig. 1).

Although the BioID workflow permits the capture of partners involved in transient PPI, this labelling can be restricted by the accessibility of BirA* ligase to lysine residues acting as biotin-acceptor sites on proximal partners^16^. Thus, we propose that the inclusion of a parallel affinity purification protocol permits a more robust and comprehensive interactome identification. For our studies, we therefore also conducted GFP-Trap pull-down of GFP-ataxin-1[85Q].

In our complementary protocols, we included strict background controls, a stress intervention and biological replicates. Specifically, for BioID analyses, samples processed from non-transfected cells reveal endogenous biotinylated proteins; for affinity purification analyses, samples processed from GFP-only transfected cells reveal binding independent of polyQ-ataxin-1. Importantly, as stress influences protein aggregation and disease development in neurodegenerative disease^21,22^, we included samples prepared from cells exposed to pro-oxidant arsenite stress. To miminize non-reproducible results in all protocols, biological triplicates were analysed. Together these approaches allow a robust identification of the ataxin-1[85Q] interactome in neuronal (Neuro-2a) cells and will drive further studies to understand how this PPI network might be targeted to ameliorate pathogenesis.

## Methods

These methods are expanded versions of descriptions in our related work^23^.

### Sample preparation

**Figure 2:**
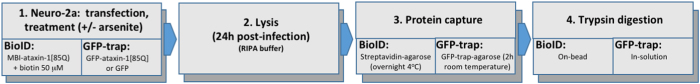
Work flow of sample preparation for mass spectrometry analysis. Following transfection to express MBI-ataxin-1[85Q], GFP-ataxin-1[85Q] or GFP only, Neuro-2a cells were incubated with/without (+/−) pro-oxidant arsenite stress. Cells were lysed, then proteins captured by streptavidin-agarose in the BIoID protocol, or by GFP-Trap. Incubation with trypsin followed on-bead (BioID) or in-solution (GFP-Trap) methods to produce peptides for further mass spectrometry analysis.

A workflow for sample preparation prior to the mass spectrometry analysis is shown in Fig. 2.

For the BioID protocol, Neuro-2a cells were transiently transfected using Lipofectamine 2000 (ThermoFisher) to express mycBioID-ataxin-1[85Q] or remained untransfected as a control; biotin (50 μM) was included during the transfection protocol. The untransfected control in this protocol allowed establishment of the endogenous biotinylation patterns in Neuro-2a cells, without restricted compartmentalization that would be expected for either cytoplasmic restricted GFP-tagged BirA* ligase or nuclear restriction if BirA* was linked to a nuclear localization sequence.

For the affinity purification protocol, subsequently called the GFP-Trap protocol, Neuro-2a cells were transiently transfected using Lipofectamine 2000 (ThermoFisher) to express GFP-ataxin-1[85Q] or GFP as a control. The GFP-transfected control in this protocol allowed establishment of the proteins interacting with GFP in the absence of ataxin-1.

After 24 h, all cells were washed with ice-cold phosphate-buffered saline (PBS) and lysed with ice-cold radioimmunoprecipitation assay (RIPA) buffer (50 mM Tris-HCl, pH 7.3, 150 mM NaCl, 0.1 mM ethylenediaminetetraacetic acid (EDTA), 1% sodium deoxycholate, 1% Triton X-100, 0.2% NaF and 100 μM Na_3_VO_4_) supplemented with cOmplete^TM^ protease inhibitor mix (Roche Diagnostic). Lysates were centrifuged (1,000 rpm, 20 min, 4 °C) and supernatants were collected. Protein concentration was determined by the BioRad protein assay, using bovine serum albumin (BSA) standards. For BioID pulldown, lysates (1.5 mg total protein) were then incubated with streptavidin-agarose (Invitrogen) overnight at 4 °C (end-over-end rotation); for GFP-Trap pulldown, lysates (1.5 mg total protein) were incubated with GFP-Trap agarose (Chromo Tek) for 2 h at room temperature (end-over-end rotation). The pellets for both protocols were captured (12,000 rpm, 1 min) and washed thoroughly (x2) using RIPA buffer. For the BioID pulldown, 5 subsequent wash steps were included: 0.5% SDS in PBS (washes 3 and 4), 6 M urea in 100 mM Tris-HCl pH 8.5 (washes 5 and 6), and 100 mM Tris-HCl pH 8.5 (wash 7). For the GFP pull-down, all 5 subsequent washes used 10 mM Tris-HCl pH 7.5, 150 mM NaCl, 0.5 mM EDTA (washes 3 to 7).

Proteins in all washed pellets were further prepared for mass spectrometry analysis. For streptavidin-agarose pellets, an on-bead digestion was performed by adding 20 μl trypsin-containing denaturing solution (50 mM urea, 5 mM tris(2-carboxyethyl) phosphine (TCEP) and 0.25 μg trypsin (Sigma) in 50 mM triethylammonium bicarbonate (TEAB)). After incubation (overnight, 37 °C), supernatants were collected. For the GFP-Trap-agarose pellets, an in-solution digestion for the eluted proteins was performed by adding the elution solution (50% aqueous 2,2,2-Trifluoroethanol (TFE)/0.05% formic acid, pH 2.0, 1 mM TCEP). After incubation (5 min, room temperature), supernatants were collected and then 5 μL trypsin solution (0.25 μg trypsin, 200 mM TEAB) added before incubation (overnight, 37 °C).

### Mass spectrometry analysis

For each trypsin-digested sample, supernatant (10 μl) was collected and tryptic peptides were analysed by liquid chromatography-MS/MS (LC-MS/MS) using an Q-Exactive plus mass spectrometer (Thermo Scientific) fitted with nanoflow reversed-phase-HPLC (Ultimate 3000 RSLC, Dionex). The nano-LC system was equipped with an Acclaim Pepmap nano-trap column (Dionex – C18, 100 Å, 75 μm × 2 cm) and an Acclaim Pepmap RSLC analytical column (Dionex – C18, 100 Å, 75 μm × 50 cm). Typically for each LC-MS/MS experiment, 1 μL supernatant was loaded onto the enrichment (trap) column (isocratic flow 5 μL/min, 3% CH_3_CN containing 0.1% formic acid, 6 min) before the enrichment column was switched in-line with the analytical column. For LC, the eluents used were 0.1% formic acid (solvent A) and 100% CH_3_CN/0.1% formic acid (solvent B) with the following sequence of gradients: 3 to 20% B (in 95 min), 20 to 40% B (in 10 min), 40 to 80% B (in 5 min), 80% B (maintained for the final 5 min) before equilibration in 3% B prior to the next analysis (10 min). All spectra were acquired in positive mode with full scan MS1 scanning from m/z 375-1400 (70000 resolution, AGC target 3e6, maximum accumulation time 50 ms, lockmass 445.120024). The 15 most intense peptide ions with charge states ≥2–5 were isolated (isolation window 1.2 m/z) and fragmented with normalized collision energy of 30 (35000 resolution, AGC target 1e5, maximum accumulation time 50 ms). An underfill threshold was set to 2% for triggering of precursor for MS2. Dynamic exclusion was activated for 30 s.

### Protein identification and background reduction

Resultant MS/MS data was analyzed using the Mascot search engine (Matrix Science version 2.4) against the SWISSPROT database released July 2015 (with the settings as follows: taxonomy – Mus musculus, enzyme - Trypsin, Protein Mass - ±20 ppm, Fragment Mass Tolerance - ±0.2 Pa, Max Missed Cleavages: 2). Identifications in all samples (test or background/non-specific binding) were accepted for proteins with >two significant peptides (p < 0.05). Background/non-specific binding proteins were identified as follows: for the GFP-Trap protocol assessments, proteins identified in samples prepared from GFP only-transfected cells processed in parallel in each of the 3 replicates were defined as background/non-specific binding; for BioID assessments, proteins identified in samples prepared from untransfected cells across all 3 replicates were pooled and defined as background as endogenously biotinylated proteins. Proteins after background reduction were retained for further biological triplicates comparison and bioinformatics analyses.

### Code availability

Data comparision was performed by Miscrosoft® Excel for Mac (Version 15.19.1) using the conditional formatting function and also by the Venn diagrams tool, available online (http://bioinformatics.psb.ugent.be/webtools/Venn/).

## Data Records

The mass spectrometry proteomics data have been deposited to the ProteomeXchange Consortium (http://proteomecentral.proteomexchange.org) via the PRIDE partner repository (Data Citation 1). The dataset includes 21 raw files, 21 mgf files, and 21 mzid files. Raw files are non-processed outputs from Q-Exactive plus mass spectrometer. Mgf files are original peak list files that were used by search engine MASCOT. Mzid files describe the results of peptide/protein identification. There are 21 samples that represent 7 conditions (Fig. 2 & Methods) (3 BioID conditions: MBI-ataxin-1[85Q] +/-Arsenite & non-transfection control; 4 GFP-Trap conditions: GFP-ataxin-1[85Q] +/-Arsenite & GFP +/-Arsenite controls) and with triplicates for each condition. Each sample has 1 raw file, 1 mgf file, and 1 mzid file that was named after the experimental condition.

## Technical Validation

**Figure 3:**
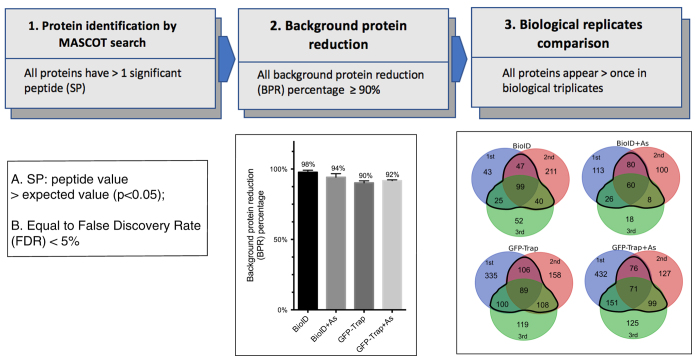
Analysis procedures for reliable and unbiased data production. Three steps were taken: Step 1 identifies all proteins with at least 2 significant peptides (SP). Significant peptides were defined as the peptide value > expected value (p <0.05) in MASCOT. With proteins having two or more SP, an FDR &<5% was achieved. Step 2 identifies background proteins in each sample and eliminates background proteins from samples data sets. Background proteins were collected from negative controls for BioID or GFP-Trap (refer to Methods for details). Background protein reduction (BPR) percentage was calculated as (number of background proteins identified in sample data set)/(number of total background proteins). Mean ± SEM are represented for each sample in the bar graph (*n* = 3). High BPR percentage (all ≥90%) indicates effective elimination of background from samples. Step 3 compares biological triplicates. For each condition, only proteins that appeared at least twice in biological triplicates were considered for further use. Proteins inside of the black contour in each venn diagram were used in final data lists.

Three steps, as illustrated in Fig. 3, were taken in technical validation to maximize data quality.

At Step 1, the protein identification stage following the MASCOT search, ion scores for each peptide were compared to ion score significance thresholds (p <0.05). Peptides with an ion score (peptide value) higher than the thresholds (expected value) were considered as significant peptides (SP). To ensure a false discovery rate (FDR) of <5%, proteins with <2 significant peptides were discarded (proteins identified by MASCOT search, Data Citation 2). This step diminished the inaccuracy caused during the mass spectrometer operation or the peptide searching/matching processes.

Step 2 was undertaken for background reduction. We included negative controls, to define background proteins: non-transfected cells to determine endogenous biotinylated proteins in BioID and GFP-transfected cells for non-specific binding in the GFP-Trap protocol. The background protein reduction (BPR) percentages for all samples were calculated as the ratio of the number of identified background proteins in each sample against the number of total background proteins (Fig. 3, bar graph). The average BPR percentage for each condition was thus calculated to be >90%, indicating an effective background reduction that identified a majority of the background proteins in each sample (proteins after background reduction, Data Citation 2).

Step 3 was performed to ensure the consistency of the results. Biological triplicates were performed and the results compared to filter lower confidence proteins. This step decreased noise caused by random biological variations in samples, enriching proteins with reproducible binding/proximity. Only proteins that appeared at least twice in triplicates in each condition were considered for further bioinformatics analyses (final proteins after biological replicates comparison, Data Citation 2).

Our results revealed 675 protein identifications in total (ataxin-1 interactome, Data Citation 2), with 91 proteins appearing three or more times in the four conditions. Our complimentary approaches have identified numerous proximal or interacting proteins of ataxin-1, including many RNA-binding proteins, reinforcing ataxin-1’s role in transcription and RNA splicing^12,24,25^. The results also show good coverage of well-defined binding partners of ataxin-1, e.g. CIC, U2AF65, and 14-3-3 proteins^9,11,12^. Importantly, these results also reveal multiple new discoveries not previously made in the single screening approaches by others; these new members of the ataxin-1 interactome thus constitute a rich resource requiring further interrogation. Thus, the employment of proximity labelling (BioID) and affinity purification (GFP-Trap) can reveal a complex interactome of polyQ-ataxin-1 protein with future applications of this combined approach possible for various other proteins, including other polyQ proteins that drive neurodegenerative diseases.

## Additional Information

**How to cite this article**: Zhang, S. *et al*. Complementary proteomics strategies capture an ataxin-1 interactome in Neuro-2a cells *Sci. Data*. 5:180262 doi: 10.1038/sdata.2018.262 (2018).

**Publisher’s note**: Springer Nature remains neutral with regard to jurisdictional claims in published maps and institutional affiliations.

## Supplementary Material
